# Intrinsic functional network organization in high-functioning adolescents with autism spectrum disorder

**DOI:** 10.3389/fnhum.2013.00573

**Published:** 2013-09-19

**Authors:** Elizabeth Redcay, Joseph M. Moran, Penelope L. Mavros, Helen Tager-Flusberg, John D. E. Gabrieli, Susan Whitfield-Gabrieli

**Affiliations:** ^1^Department of Psychology, University of Maryland, College ParkMD, USA; ^2^Center for Brain Science, Harvard UniversityCambridge, MA, USA; ^3^United States Army Natick Soldier Research Development and Engineering CenterNatick, MA, USA; ^4^Simons Center for the Social Brain at Massachusetts Institute of TechnologyCambridge, MA, USA; ^5^Department of Psychology, Boston UniversityBoston, MA, USA; ^6^McGovern Institute for Brain Research at Massachusetts Institute of TechnologyCambridge, MA, USA; ^7^Department of Brain and Cognitive Sciences, Massachusetts Institute of TechnologyCambridge, MA, USA

**Keywords:** autism, resting-state functional connectivity, default mode network, intrinsic network organization, graph theory, functional MRI

## Abstract

Converging theories and data suggest that atypical patterns of functional and structural connectivity are a hallmark neurobiological feature of autism. However, empirical studies of functional connectivity, or, the correlation of MRI signal between brain regions, have largely been conducted during task performance and/or focused on group differences within one network [e.g., the default mode network (DMN)]. This narrow focus on task-based connectivity and single network analyses precludes investigation of whole-brain intrinsic network organization in autism. To assess whole-brain network properties in adolescents with autism, we collected resting-state functional connectivity MRI (rs-fcMRI) data from neurotypical (NT) adolescents and adolescents with autism spectrum disorder (ASD). We used graph theory metrics on rs-fcMRI data with 34 regions of interest (i.e., nodes) that encompass four different functionally defined networks: cingulo-opercular, cerebellar, fronto-parietal, and DMN (Fair etal., 2009). Contrary to our hypotheses, network analyses revealed minimal differences between groups with one exception. Betweenness centrality, which indicates the degree to which a seed (or node) functions as a hub within and between networks, was greater for participants with autism for the right lateral parietal (RLatP) region of the DMN. Follow-up seed-based analyses demonstrated *greater* functional connectivity in ASD than NT groups between the RLatP seed and another region of the DMN, the anterior medial prefrontal cortex. Greater connectivity between these regions was related to lower ADOS (Autism Diagnostic Observation Schedule) scores (i.e., lower impairment) in autism. These findings do not support current theories of underconnectivity in autism, but, rather, underscore the need for future studies to systematically examine factors that can influence patterns of intrinsic connectivity such as autism severity, age, and head motion.

## INTRODUCTION

Atypical patterns of functional and structural connectivity are proposed to be a hallmark neurobiological feature of autism (Belmonte et al., 2004; Just et al., 2004; Courchesne and Pierce, 2005; Cherkassky et al., 2006). Most theories and data point to a pattern of underconnectivity, particularly for long-distance connections such as interhemispheric or anterior–posterior intrahemispheric connections (Belmonte et al., 2004; Just et al., 2004; Anderson et al., 2011; Dinstein et al., 2011). Some also suggest an increase in local connections at the expense of long-distance connections (Courchesne and Pierce, 2005; Courchesne et al., 2007; Rippon et al., 2007). Recent findings, however, offer mixed support and suggest a more complex picture of connectivity differences in autism with evidence for both hypo- and hyper-connectivity for short- and long-distance connections, depending partly on the specific experimental and analytic methods used and age of the participants (e.g., Courchesne et al., 2007; Noonan et al., 2009; Khan et al., 2013; Lynch et al., 2013; review, Müller et al., 2011).

Structural connectivity findings, indexed by measures of white matter integrity from diffusion tensor imaging (DTI) (e.g., fractional anisotropy, or FA) or white matter volumes from structural MRI, reveal atypical connectivity patterns in autism but do not support general underconnectivity in autism. Rather, findings suggest developmentally *increased* white matter volume (Courchesne et al., 2001; Hazlett et al., 2006), particularly radiate white matter bundles supporting interhemispheric and cortico-cortical connections (Herbert et al., 2004) and increased FA in infants and young children with autism (e.g., Ben Bashat et al., 2007; Wolff et al., 2012), whereas later in development (e.g., adolescents and adults), FA is decreased (e.g., Barnea-Goraly et al., 2004; Lee et al., 2007; Nair et al., 2013).

Studies of *functional* connectivity, or the correlation in signal between brain regions, largely have supported the underconnectivity theory when functional connectivity has been assessed in the context of a task (review, Müller et al., 2011). This pattern of reduced long-distance connectivity (e.g., between regions of different hemispheres or lobes) is seen across domains of function including tasks involving language processing (e.g., Just et al., 2004; Kana et al., 2006), executive function (e.g., Just et al., 2007), and social processing (e.g., Mason et al., 2008; Kana et al., 2012; , but see Murphy et al., 2012), but notably these tasks also resulted in reduced activation in the autism spectrum disorder (ASD) group as compared to the neurotypical (NT) group. Thus, while informative, task-based functional connectivity analyses may reflect differences in performance during a task and may not reflect differences in intrinsic functional organization of the brain.

Task-independent studies of the “resting” brain provide a window with which to examine intrinsic functional network organization. As first noted by Biswal et al. (1995), even in the absence of a specific task, fluctuations in brain signal are temporally correlated within regions that are part of the same functional network. These large-scale functional networks can be identified using data-driven ICA (independent component analysis) analyses (e.g., Damoiseaux et al., 2006) or seed-based analyses (e.g., Fox et al., 2005) and are thought to reflect regions that have a history of co-activation. Indeed, differences in the organization or connection strength within these regions are related to developmental changes (e.g., Fair et al., 2009), training (Lewis et al., 2009), and individual differences, for example in memory (Wang et al., 2010), math abilities (Emerson and Cantlon, 2012), and face processing (Zhu et al., 2011), suggesting intrinsic network connectivity is behaviorally relevant.

There has been considerable divergence across studies in regards to the status of resting-brain functional connectivity in ASD. Like task-based studies, many studies of the resting brain in ASD (or those in which the task is used as a regressor of no interest) have revealed reduced functional connectivity in ASD, particularly for long-range connections (Cherkassky et al., 2006; Kennedy and Courchesne, 2008; Ebisch et al., 2011; Tsiaras et al., 2011; Murdaugh et al., 2012; Rudie et al., 2012; Washington et al., 2013). However, unlike task-based studies, a number of studies report findings that are inconsistent with a general theory of underconnectivity (e.g., Monk et al., 2009; Müller et al., 2011; Tyszka et al., 2013), and in some cases hyper-connectivity in ASD groups has been reported (Mizuno et al., 2006; Turner et al., 2006; Noonan et al., 2009; Di Martino et al., 2011; Shih et al., 2011; Lynch et al., 2013).

In sum, extant data suggest a general underconnectivity theory in autism is likely not the full story. Possibly, the age of the participant, the context in which connectivity is assessed (e.g., resting vs. task), and the specific networks examined may result in different findings between groups. Further, recent studies suggest that head motion may lead to systematic, spurious correlations which could mimic some of the same patterns of connectivity differences reported between autism and NT groups (Power et al., 2011). An incomplete picture of how each of these factors contributes to functional connectivity in autism still remains. One additional contributing factor is that most previous studies only focused on the strength of correlations within a single network rather than examining network organization with graph theoretical metrics. Recent advances in graph theory (or complex network) analyses for resting-state functional connectivity MRI (rs-fcMRI) data allow for characterization of whole-brain intrinsic network organization (e.g., review, Rubinov and Sporns, 2010; Bullmore and Bassett, 2011). Specifically, rather than focusing on the strength of region–region correlations, graph theory methods can examine the topological properties of each region within the context of all other regions of interest. For example, graph theory metrics can include measures of the integration (global efficiency, average path length), segregation (local efficiency, clustering coefficient), and centrality (betweenness centrality) of networks. Thus, these metrics can provide a more robust test of the theory of reduced long-distance and increased local connectivity by testing differences in measures of whole-brain network integration and segregation.

In the current study, we assessed whole-brain network properties in a group of adolescents with and without autism by using graph theory and seed-based analyses on rs-fcMRI data with functionally defined regions of interest. The functional regions of interest included 34 regions identified from previous meta-analyses (Dosenbach et al., 2006; Fair et al., 2009) that encompass four different functionally defined networks: cingulo-opercular (CO), cerebellar (C), fronto-parietal (FP), and default mode (DMN; Fair et al., 2009). These networks were chosen because previous research with these same networks has demonstrated a developmental pattern of progressive increases in long-distance connectivity between nodes of the same network and concurrent decreases in connectivity between anatomically proximal nodes of distinct networks (Fair et al., 2008, 2009). Furthermore, functions associated with these networks have all been implicated in autism (e.g., reviews, Di Martino et al., 2009; Minshew and Keller, 2010). Thus, examining these networks allows for a more rigorous test of the hypothesis of reduced long-distance and increased local connectivity in autism, across multiple networks that support varied functions.

## MATERIALS AND METHODS

### PARTICIPANTS

All participants gave written, informed consent and parental consent was obtained for participants under 18 years of age as approved by the Committee on the Use of Humans as Experimental Subjects (COUHES) at the Massachusetts Institute of Technology. Participants were compensated monetarily for their time. Participants were part of a multi-site study involving three visits for TD adolescents and four for the ASD group but only the resting-state functional MRI data are presented in the current study. Participant IQ was measured using the Kaufman Brief Intelligence Test (KBIT-2).

### AUTISM SPECTRUM DISORDER PARTICIPANTS

We collected resting-state functional MRI data from 22 male adolescents and young adults (14–20 years; mean 17.3 ± 2.2 years; all male) with a clinical diagnosis of ASD or Asperger’s disorder. Diagnosis was confirmed using a combination of the Autism Diagnostic Observation Schedule (ADOS) Module 3 or 4 (administered to the participant; Lord et al., 2000) and the Social Communication Questionnaire (SCQ; completed by the parent of the participant; Corsello et al., 2007). The SCQ is a questionnaire designed to screen for autism and all included ASD participants received an SCQ score greater than the suggested cut-off for ASD of 15 (mean 21.6; 16–28). All participants reached criteria for Autism or spectrum from the ADOS except 1 who was subsequently removed from the analyses. Seven participants were excluded from the analyses because of excessive movement artifact (see below for description) resulting in a final sample of 14 participants with ASD (**Table 1**). Information about co-morbid diagnoses and current medications were obtained through a phone screen with either the participant or parent if the participant was a minor. This information was not available for 2 of the 14 ASD participants. Six of the 12 participants reported use of medications associated with symptoms of neuropsychiatric disorders [ADHD (4), depression/anxiety (3), psychosis (2)]. Only two participants, however, reported any co-morbid neurological disorders and these were obsessive–compulsive disorder (1) and attention deficit hyperactivity disorder (2).

**Table 1:** Demographic and head motion information for NT andASD groups and thoseASD participants excluded due to excessive head motion.

	NT (*N* = 14)	ASD (*N* = 14)	ASD-excluded (*N* = 7)	NT vs. ASD (*p*-value)	ASD vs. ASD-excluded (*p*-value)
Age	17.7(1.8)	17.8(1.9)	15.8(2.5)	0.81	0.05
Full Scale IQ	119(9.6)	116.9(13.7)	98.3(24.4)	0.59	0.04
Verbal IQ	118(13.1)	116.3(15.1)	97(24.9)	0.75	0.04
Non-verbal IQ	115(10.3)	112.5(13.1)	99.7(27.3)	0.57	0.17
Motion outliers	2.2(3.8)	1.8(2.8)	45.9(17.3)	0.73	<0.0001°
ADOS Combined	N/A	9.5(1.3)	16.2(2.8)	N/A	0.02
ADOS Comm.	N/A	3(2)	4.2(2.3)	N/A	0.28
ADOS Social	N/A	6.5(2.8)	12(5.1)	N/A	0.007

*Note: Data are mean (SD). Age is in years. IQ was measured using the Kaufman Brief Intelligence Test-2. ADOS Comm is the communication subscale. *p*-Value is based on a *t*-test comparing groups. °This difference is circular because these groups were created based on differences in motion outliers.*

### NEUROTYPICAL PARTICIPANTS

Twenty-three NT participants (14–20 years; all male) performed a resting-state scan. Participants were excluded if they reported any psychiatric or neurological disorders on a self-report screening questionnaire, which was filled out either by the participant or the parent. To screen for the presence of autism or autistic-like traits in the typical population, the participant’s parents completed the SCQ screening described above. One participant who was no longer a minor completed the Autism Spectrum Quotient (AQ; Woodbury-Smith et al., 2005). No included participants received scores above the suggested threshold for autism screening. One was excluded due to excessive movement. Of the 22 remaining participants, 14 were matched as closely as possible to the ASD group on age. IQ scores did not differ significantly between groups (see **Table 1**).

### MRI DATA ACQUISITION

Participants came to the Athinoula A. Martinos Imaging Center at the McGovern Institute for Brain Research at MIT for MRI data collection on a 3T Siemens Magnetom Tim Trio Scanner. We collected a structural MPRAGE image (128 sagittal slices, TE = 3.39 ms, TR = 25 ms, voxel size 1.3 mm × 1 mm × 1.3 mm) and a resting-state functional MRI scan (67 sagittal slices, TE = 30 ms, TR = 6000 ms, # of TRs = 64, voxel size = 2.0 mm isotropic) as part of a 90-min battery of tasks examining social processing that are not presented here. The last scan of the battery was the resting-state scan for which we asked participants to remain still with eyes open and fixated on a cross in the center of the screen. We chose a 6 s TR for the resting-state scan in order to achieve high spatial resolution with whole-brain coverage because previous work has demonstrated that array coils provide the biggest increases in temporal signal to noise ratio (tSNR) at high spatial resolutions (Triantafyllou et al., 2011). While this TR is unusually long, a study by Van Dijk et al. (2010), showed that there was no significant difference in the correlation strengths between the resting-state networks when compared between a TR of 2.5 and 5 s.

### FUNCTIONAL MRI PREPROCESSING

All data were analyzed using SPM8^1^, Nipype (Gorgolewski et al., 2011), the CONN functional connectivity toolbox ver 13e^2^ (Whitfield-Gabrieli and Nieto-Castanon, 2012), and in-house Matlab (The Mathworks, Natick, MA, USA) scripts. All resting-state volumes were corrected for differences in the timing of slice acquisition. Functional data were realigned to the mean of all functional volumes in the timeseries using a 6° rigid spatial transformation, which provided the spatial deviation for each timepoint for translational (x, y, z) and rotational (roll, pitch, yaw) directions of movement. Functional data were then smoothed with a Gaussian smoothing kernel of 6 mm full-width half maximum, and normalized into standard Montreal Neurological Institute (MNI) space using non-linear transformations.

### ANALYSES OF HEAD MOTION

The artifact detection toolbox (ART)^3^ was used to examine outliers in global signal and movement for each participant. Timepoints were marked as outliers if global signal exceeded three standard deviations of the mean or if movement exceeded 1 mm (across translational and rotational directions) of scan-to-scan deviation. Participants for whom greater than 20% of the run was marked as an outlier were removed from the analyses (seven ASD; one NT). Head motion has been shown to result in spurious patterns of correlations (both increased and decreased; e.g., Power et al., 2011). Thus to examine whether groups differed as a function of head motion we used between-group *t*-tests to test for differences in (1) the total number of outliers and (2) the sum across all volumes of the absolute value of the deviation (in mm) from the reference volume (i.e., the realignment parameters) for each of the six possible motion directions (i.e., x, y, z, roll, pitch, yaw). Using between group *t*-tests, we also examined whether those participants who were excluded from the analyses due to excessive head motion were systematically different from those included in terms of age, IQ, or autism severity (**Table 1**). No significant differences in head motion between groups were present for either the number of outliers (see **Table 1**) or realignment parameters in any of the six directions [x: *t*(24) = -0.56, *p* < 0.58; y: *t*(24) = -0.58, *p* < 0.57; z: *t*(24) = 1.1, *p* < 0.28; roll: *t*(24) = 0.85, *p* < 0.41; pitch: *t*(24) = 0.18, *p* < 0.86, yaw: *t*(24) = 1.7, *p* < 0.11). However, the ASD participants who were excluded due to excessive head motion had significantly lower Verbal Composite IQ scores, and higher (worse) social impairments as measured by the ADOS Reciprocal Social Interaction subscale and autism severity as measured by the Combined ADOS Communication and Reciprocal Social Interaction subscales. Excluded participants also showed a trend toward significantly younger ages (**Table 1**).

### FUNCTIONAL CONNECTIVITY ANALYSES

To minimize the effects of head motion, whole-brain voxel-wise regression analyses were run for each seed region of interest with the six motion parameters from realignment and their temporal derivatives and each outlier timepoint entered separately as noise covariates. Additionally, using the aCompCor method (Behzadi et al., 2007) to account for physiological noise, covariates were included with a principal components analysis (PCA)-reduction (three dimensions) of the signal from white matter and CSF voxels based on each individual’s unique segmented white matter and CSF masks. The residual datasets were then temporally filtered (0.01 < *f* < 0.08) to focus analyses to the low-frequency oscillations characteristic of resting-state networks.

Whole-brain regression analyses were computed for each of the 34 seed regions of interest (Fair et al., 2009; **Table 2**) on the preprocessed, “clean” datasets for each participant. These analyses resulted in a correlation value in each voxel for each of the 34 seed regions. Normalized correlation values were created by a Fishers r-to-z transform and used in subsequent analyses. Averaging the normalized correlation coefficients within each group for each region pair created correlation matrices for each of the 34 regions of interest (ROI). Two-way between group (ASD vs. NT) *t*-tests were run for each of the 561 ROI–ROI pairs to examine whether differences in connectivity strength between groups were present and specific to particular networks. False discovery rate (FDR; *q* < 0.05) was used to correct for multiple comparisons for the ROI–ROI comparisons.

**Table 2:** Seed regions of interest.

Number	Network	Hemi	Region	X	V	Z
1	Cingulo-opercular	L	Anterior prefrontal cortex anterior insula/frontal	-28	51	15
2	Cingulo-opercular	L	Operculum	-35	14	5
3	Cingulo-opercular	L	Anterior thalamus dorsal anterior cingulate/medial superior	-12	-15	7
4	Cingulo-opercular	L	Frontal cortex	-1	10	46
5	Cingulo-opercular	R	Anterior prefrontal cortex anterior insula/frontal	27	50	23
6	Cingulo-opercular	R	Operculum	36	16	4
7	Cingulo-opercular	R	Anterior thalamus	10	-15	8
8	Cerebellar	L	Inferior cerebellum	-19	-78	-33
9	Cerebellar	L	Lateral cerebellum	-32	-66	-29
10	Cerebellar	R	Inferior cerebellum	18	-80	-33
11	Cerebellar	R	Lateral cerebellum	31	-61	-29
12	Default	L	Inferior temporal	-61	-33	-15
13	Default	L	Lateral parietal	-47	-67	36
14	Default	L	Parahippocampal gyrus	-22	-26	-16
IS	Default	L	Superior fronta	-14	38	52
16	Default	R	Inferior temporal	65	-17	-15
17	Default	R	Lateral parietal	53	-67	36
18	Default	R	Parahippocampal gyrus	25	-26	-14
19	Default	R	Superior frontaanterior medial prefrontal	17	37	52
20	Default	R	Cortex	1	54	21
21	Default	L	Posterior cingulate cortex	-2	-36	37
22	Default	R	Retrosplenial cortex ventromedial prefrontal	3	-51	8
23	Default	L	Cortex	-3	39	-2
24	Fronto-parietal	L	Inferior parietal lobe	-51	-51	36
25	Fronto-parietal	L	Intraparietal sulcus	-31	-59	42
26	Fronto-parietal	L	Dorsolateral prefrontal cortex	-43	22	34
27	Fronto-parietal	L	Frontal	-41	3	36
28	Fronto-parietal	L	Precuneus	-9	-72	37
29	Fronto-parietal	R	Inferior parietal lobe	51	-47	42
30	Fronto-parietal	R	Intraparietal sulcus	30	-61	39
31	Fronto-parietal	R	Dorsolateral prefrontal cortex	43	22	34
32	Fronto-parietal	R	Frontal	41	3	36
33	Fronto-parietal	R	Precuneus	10	-69	39
34	Fronto-parietal	LR	Mid cingulate cortex	0	-29	30

**FIGURE 1:**
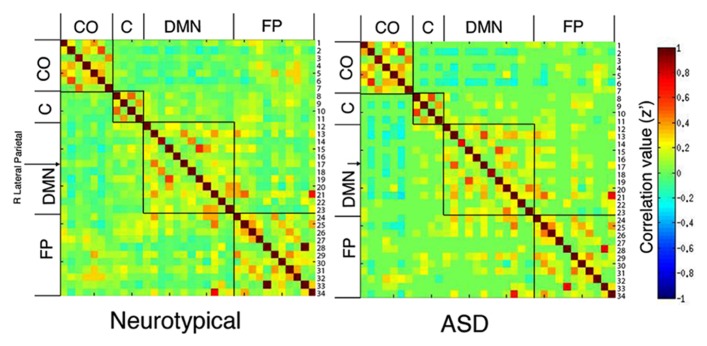
**Correlation matrices for neurotypical (A) and ASD (B) groups.** Normalized correlation coefficients are reported for each of the 34 × 34 ROI correlations by group. These are organized by network based on Fair et al. (2009) (CO, cingulo-opercular; C, cerebellar; DMN, default mode network; FP, fronto-parietal). Each row is labeled with a number which corresponds to 1 of 34 seed regions (see **Table 2** for a list by number). Comparison of these matrices resulted in no significant differences between groups, when corrected for multiple comparisons. The right lateral parietal seed region (#17) of the DMN is identified with an arrow because that region showed a significant effect of group on centrality measures.

*These regions of interest and coordinates are taken directly from Fair et al. (2009). Number corresponds to the number listed in **Figure 1**.*

Graph theory analyses were computed using the CONN functional connectivity toolbox. The unweighted ROI-to-ROI correlation matrices were first thresholded at a cost value of *k* = 0.15. Cost is a measure of the proportion of connections for each ROI in relation to all connections in the network. Rather than determining a fixed correlation value as a threshold (e.g., *r* = 0.1), using a cost threshold allows for roughly the same number of connections across participants by varying the correlation threshold for each participant to achieve the fixed cost threshold. When cost is equated across participants, direct comparisons across groups of network property differences can be made. Small world properties are observed in the range of costs 0.05 < *k* < 0.34, where global efficiency is greater than that of a lattice graph and local efficiency is greater than that of a random graph (Acha2007rd and Bullmore, 2007). A cost threshold of .15 has also been demonstrated to provide a high degree of reliability when comparing session-specific estimates of graph theoretical measures across repeated runs or sessions (e.g., global efficiency *r* = 0.95, local efficiency *r* = 0.9; Whitfield-Gabrieli and Nieto-Castanon, 2012). We employed both one- and two-sided cost thresholds. In a one-sided cost threshold only positive correlations are considered, whereas two-sided includes both positive and negative correlations. To confirm that our findings generalize beyond these specific parameters, data were examined at a cost threshold of 0.05, 0.1, 0.2, and 0.25 and compared to the findings with our *a priori* cost threshold of 0.15.

The specific measures of interest were those of integration (global efficiency), segregation (local efficiency), and centrality (betweenness centrality). Between-group *t*-tests were used to compare network measures between groups with a FDR correction of *q* < 0.05. Global efficiency is calculated as the average of the inverse of the shortest path length between each ROI (or node) and all other ROIs. The shortest path length is defined as the fewest number of connections (or correlations) between two nodes. Thus, a network with high global efficiency would be one in which nodes are highly integrated so the path between nodes is consistently short. With cost kept constant, this measure can be thought of as reflecting global, long-distance connections within the brain. Local efficiency is calculated as the average inverse of the shortest path length between the neighbors of any given node (or ROI). In other words, local efficiency measures the extent to which nodes are part of a cluster of locally, interconnected nodes. Finally, we examined a measure of centrality, betweenness centrality, which measures the fraction of all shortest path lengths in a network that pass through a given node. Thus, if a node is directly connected to many other nodes in the network it will have a shorter overall path length and function as a hub within and between networks. For more details on graph theoretical measures see Bullmore and Bassett (2011) or Rubinov and Sporns (2010).

## RESULTS

### LARGELY TYPICAL NETWORK ORGANIZATION IN ASD

Comparison of normalized correlation matrices between groups revealed minimal differences, which do not survive correction for multiple comparisons. Similarly network analyses revealed largely typical patterns of connectivity in the ASD group as compared to the NT group. Contrary to our hypotheses we found no differences in measures of global or local efficiency. Only betweenness centrality, which indicates the degree to which a seed (or node) functions as a hub within and between networks, was significantly different between groups and it was *greater* for participants with autism for the right lateral parietal (RLatP) seed of the DMN (*t*(26) = 3.52; *p* < 0.027 FDR-corrected) only. This metric was only significantly different when both positive and negative correlations were used in the cost threshold. When only positive correlations were considered, greater betweenness centrality in RLatP remained larger in ASD than NT groups but not significantly (*t*(26) = 1.57, *p* < 0.13). This finding suggests both correlations and anti-correlations (i.e., negative correlations) drove differences between groups. This effect held when examining higher cost thresholds (*k* = 0.2 and 0.25) but not lower (*k* = 0.1 and 0.05).

### EXPLORATION OF RIGHT LATERAL PARIETAL SEED CONNECTIVITY PATTERNS

**FIGURE 2:**
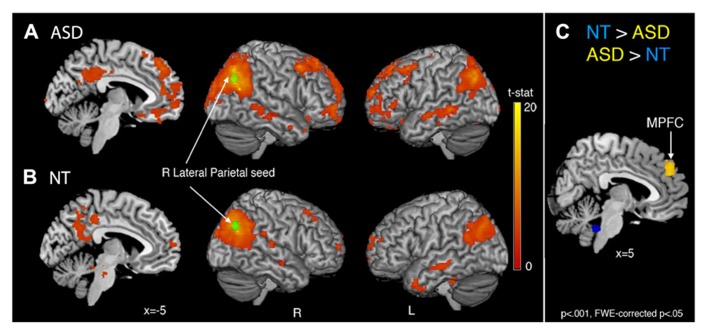
**Whole-brain functional connectivity maps with the right lateral parietal (RLatP) region (green) as a seed region are shown for the ASD group (A) and neurotypical group (B).** Between-group comparisons **(C)** revealed one region of significantly greater connectivity from the RLatP seed in the ASD than NT group (yellow) which was the medial prefrontal cortex. The NT group showed greater connectivity between the RLatP seed and regions within the cerebellum (blue) than the ASD group. All maps are thresholded at *p* < 0.001, FWE cluster corrected at *p* < 0.05.

Comparison of the 34 × 34 matrix of normalized correlation values between seed regions for each group suggests the higher betweenness centrality in ASD may be due to (1) greater long-distance connectivity within the default mode network [RLatP–anterior medial prefrontal cortex (aMPFC)] and (2) greater negative correlations with regions in cerebellar and control networks in participants with ASD (**Figure 1**). However, these ROI-to-ROI differences were not significant when controlling for multiple comparisons. To further investigate how differences in connectivity resulted in the difference in centrality between groups we conducted within- and between-group *t*-tests on correlation maps using the RLatP region as a seed region (**Figure 2**, **Table 3**). These maps demonstrate significantly *greater* functional connectivity in the ASD than NT group within medial prefrontal cortex using a FWE cluster correction of *p* < 0.05. The NT group showed higher connectivity between the RLatP seed and cerebellar tonsils [a region previously associated with the default mode network (Fox et al., 2005)]. Examination of correlation maps within each group suggests these regions of between-group differences are not driven only by negative correlations in one group.

**Table 3:** Functional connectivity from the right lateral parietal seed region.

Group region	Hemi	X	y	z	T	k
**Neurotvpical**
Lateral parietal lobe	R	50	-70	36	21.28	2818
Posterior cingulate	L	-2	-34	46	7.76	2091
Lateral parietal lobe	L	–38	-74	40	8.74	1321
Parahippocampal gyms	L	–12	-36	-16	6.54	319
Superior temporal sulcus	R	–46	-32	-6	5.77	293
Superior frontal gyrus	L	–38	54	22	5.46	225
Middle frontal gyrus	R	26	32	40	6.87	210
Posterior insula	R	38	-28	16	6.06	196
anterior superior temporal sulcus	L	–50	10	-34	5.78	85
Superior frontal gyrus	R	28	66	10	6.06	81
Cerebellar tonsils	R	10	-40	-38	6.03	75
Parahippocampal gyrus	R	26	-30	-14	6.08	72
Brainstem/pons	R	2	-28	-26	8.06	65
**Autism spectrum disorder**
Superior frontal gyrus	R	38	14	50	11.65	7006
Lateral parietal lobe	R	50	-62	22	18.56	3246
Lateral parietal lobe	L	–42	-74	38	11.95	1867
Posterior cingulate	R	12	-26	34	9.23	1710
Superior temporal sulcus	L	–54	-40	-4	10.29	552
Superior temporal sulcus	R	46	-36	-6	6.65	263
Superior frontal gyrus	L	40	58	0	8.41	211
**Neurotvpical > autism spectrum disorder**
Cerebellum	L	–24	-40	-50	5.09	127
Cerebellar tonsils	R	8	-42	-38	5.65	123
**Autism spectrum disorder > neurotypical**
Anterior medial prefrontal cortex	R	6	46	30	4.26	151

*Regions were identified using *p* < 0.001, and FWE-cluster-correction of *p* < 0.05. Coordinates are given in MNI space. *T*-values from the peak voxel of the cluster and size (*k*) of the cluster are given. Clusters are organized by size.*

**FIGURE 3:**
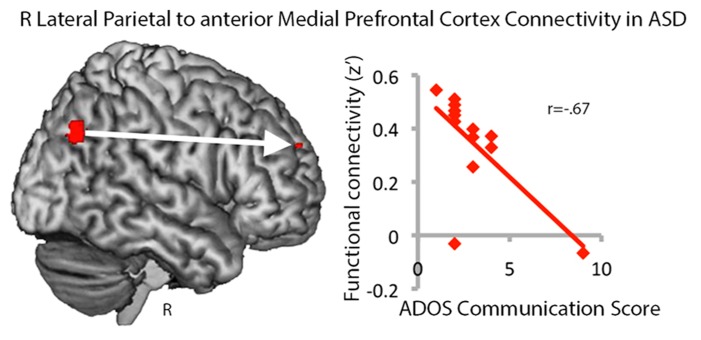
**Functional connectivity between the right lateral parietal and anterior medial prefrontal cortex regions in the default mode network is negatively correlated with ADOS communication scores in the ASD group.** Higher scores indicate greater impairment.

Our findings of greater connectivity within long-distance regions of the default mode network and greater centrality in autism were surprising and thus we explored whether variance in RLatP connectivity was related to autism severity, as measured by the ADOS, IQ, or age. No significant relationships were seen for autism severity or IQ and betweenness centrality measures for the RLatP, although there was a trend toward *reduced* centrality with age in the ASD group only [*r*(13) = -0.48, *p* < 0.086). Because the aMPFC was a region that showed significantly increased connectivity with RLatP in ASD in whole-brain analyses, we examined whether the strength of connectivity between the RLatP seed and the aMPFC seed was correlated with ADOS scores, IQ, or age. We found a negative correlation between the ADOS combined social-communication subscale and RLatP to aMPFC connectivity [*r*(13) = -0.56, *p* < 0.046), which was driven by the communication subscale [*r*(13) = -0.67, *p* < 0.012), suggesting lower connectivity within long-distance regions of the default mode network is related to more severe autism (**Figure 3**). No other correlations reached significance.

## DISCUSSION

Overall, these data are consistent with recent studies suggesting largely typical patterns of functional connectivity in individuals with autism (Tyszka et al., 2013). Although network organization across four functional networks was examined, this relatively high-functioning group of adolescent males demonstrated only one significant difference in graph theoretical metrics of network organization: namely, betweenness centrality of the RLatP region of the DMN. Follow-up whole-brain voxel-wise analyses with the RLatP region as a seed region revealed *greater *connectivity in ASD to another region of the DMN, the aMPFC, as compared to NT controls.

Of the four functional networks examined in the current study, the DMN is the most consistently implicated in autism – though that may be largely due to a bias in the number of studies investigating this network alone. The DMN comprises a set of regions showing *deactivation* during goal-directed tasks, higher metabolic activity during rest, and relative activation during tasks requiring internally directed thought or social processing (e.g., Gusnard and Raichle, 2001). In autism, however, these regions do not show the typical pattern of deactivation during goal-directed tasks (Kennedy et al., 2006; Murdaugh et al., 2012) and show reduced activation during tasks of social-cognitive processing (e.g., Gilbert et al., 2009; Murdaugh et al., 2012, but see Dufour et al., in press). Furthermore, many previous studies have found a pattern of *reduced* DMN functional connectivity in ASD, particularly between long-distance frontal and parietal regions (Kennedy and Courchesne, 2008; Monk et al., 2009; Assaf et al., 2010; Weng et al., 2011; Murdaugh et al., 2012; Rudie et al., 2012; von dem Hagen et al., 2013, but see Lynch et al., 2013). Thus, while findings of atypical engagement of the DMN in autism is not new, the finding of *greater* functional connectivity between RLatP and medial prefrontal regions of the default mode network in ASD is inconsistent with many previous studies.

There are (at least) two factors that may account for differences between our study and previous studies finding reduced connectivity between groups. First, we matched groups on head motion parameters and used two measures to account for uncorrected head motion in subsequent analyses. While some previous studies demonstrated no significant differences in head motion between groups, four of the seven studies that showed reduced functional connectivity in the DMN did not compare head motion across groups. Differences in head motion between groups is a critical factor as previous studies have suggested that head motion may account for systematic and spurious correlations, particularly in reducing long-distance correlations while increasing short-distance correlations (Power et al., 2011). It remains unclear if “accounting” for head motion in the analysis is sufficient to eliminate group differences that may be due to motion.

Second, our final sample consisted of quite high-functioning individuals with autism. Many previous studies reporting reduced functional connectivity had, on average, slightly higher ADOS scores and lower IQs. Further, within the current study a significant relationship was found between functional connectivity between RLatP and MPFC and ADOS combined social-communication (and communication) scores, with greater impairment relating to lower functional connectivity. Taken together, these findings suggest lower-functioning autism may result in patterns of reduced connectivity. However, we offer caution in this interpretation because this relationship is counter-intuitive in the context of the current study. The ASD group had significantly greater connectivity than the NT group, which suggests that more severe autism should be related to greater connectivity, but instead the reverse is true. These data suggest a possible non-linear relationship between autism severity and functional connectivity in autism but this has yet to be systematically examined.

Systematically examining how level of functioning impacts connectivity patterns is especially challenging because lower-functioning individuals tend to have more motion artifact, and, as discussed above, head motion differences alone can lead to a pattern of reduced long-distance connectivity. In the current study, we used stringent criteria to exclude participants with excessive head motion and while this only resulted in loss of data from one NT participant, seven participants with ASD were removed from data analyses. These seven were significantly different from the rest of the ASD group not only because they moved more during the scan but also because they were younger, had higher ADOS scores (i.e., were more impaired), and had lower verbal and composite IQ scores. Thus, a significant, but necessary, challenge for further research is to characterize the functional significance of resting-state networks when head motion is equated across groups (Deen and Pelphrey, 2012), such as in the current study.

Although less common, this is not the first study to report hyper-connectivity within the default mode network in autism. Two previous studies also reported increased connectivity in ASD within default mode regions (Monk et al., 2009; Lynch et al., 2013), and for one (Lynch et al., 2013) this increased connectivity was found between frontal and parietal DMN regions similar to the current study. Specifically, Lynch et al. (2013) examined functional connectivity from regions within posteromedial cortex in 7–12-year-old children and reported greater connectivity in ASD from retrosplenial cortex, a region just inferior to the posterior cingulate and part of the default mode network, to several other regions including the aMPFC (though this particular connection was reduced in the ASD sample in Monk et al., 2009). Additionally, connectivity between posterior cingulate and several lateral and medial temporal regions showed greater connectivity in the ASD than NT groups – a finding similar to Monk et al. (2009).

The study of Lynch et al. (2013) was among the first to examine DMN connectivity during a resting baseline in young children with ASD. As such, they suggested the relatively novel finding of hyper-connectivity within the default mode network (and from posteromedial cortex to regions outside of the DMN) may be due to a developmental change in the pattern of connectivity differences between ASD and NT groups. This developmental story is consistent with other theories of connectivity in autism (e.g., Courchesne and Pierce, 2005; Pelphrey et al., 2011) as well as evidence of age-related changes in brain differences between autism and control groups (Redcay and Courchesne, 2005). In other words, whereas findings from older children and adults reveal reduced brain size, reduced measures of white matter integrity (e.g., FA) or reduced functional connectivity, findings from younger children reveal larger brain size (e.g., Courchesne et al., 2001; Hazlett et al., 2006), higher FA values (Wolff et al., 2012), and increased functional connectivity (Lynch et al., 2013). However, the current findings of DMN hyper-connectivity was in a sample of adolescents and the Monk et al. (2009) study was in adults. Thus, age-related differences may not completely account for patterns of increased functional connectivity within the default mode network.

While further research is needed to disentangle the factors contributing to relatively typical or *increased *connectivity in autism, we find the increased connectivity between the RLatP and aMPFC regions of the DMN in the current study intriguing. These regions play an important role in social processes that are atypical in individuals with autism, including mental state judgments of others (i.e., theory of mind) and of one’s self (i.e., introspection) (e.g., Baron-Cohen et al., 1985; Frith and Happe, 1999; Saxe and Kanwisher, 2003; Saxe et al., 2006; Senju et al., 2009). While the medial prefrontal cortex plays a general role in mentalizing (Whitfield-Gabrieli et al., 2011), portions of RLatP cortex may play a more specific role in thinking about others thoughts and beliefs, or theory of mind (e.g., Saxe and Kanwisher, 2003; Saxe et al., 2006). Meta-analyses suggest the RLatP region of the default mode is at least partially overlapping with the right temporoparietal junction (RTPJ) often reported in studies of theory of mind processing (e.g., Schilbach et al., 2008; Spreng and Mar, 2012). Beyond social-cognitive processing, the RLatP lobe is also associated with shifts of spatial attention (Corbetta and Shulman, 2002), semantic processing (Binder et al., 1999), and narrative comprehension (e.g., Mar, 2011), all of which have been implicated as atypical in individuals with autism. Thus, greater connectivity within right parietal cortex could indicate less functional specialization of this region in ASD, similar to findings of right posterior temporal cortex (e.g., Shih et al., 2011). However, the current data do not directly address that hypothesis.

A notable limitation in this study, which claims minimal differences in functional connectivity between groups, is a small sample size. Nonetheless, the current findings of *greater* connectivity within the DMN in ASD adds to the small, growing body of literature suggesting inconsistent support for an underconnectivity theory of autism. A second limitation is the restricted range of high-functioning participants with autism who were able to complete the scan with minimal motion artifact. Even within this narrow range, a correlation was seen between a greater level of communicative impairment and lower functional connectivity between RLatP and medial prefrontal cortex and a trend toward increasing age and reduced betweenness centrality in ASD. Finally, a third limitation is the inclusion of data from participants currently on medication as some medications may affect the strength or patterns or brain activation; however, the sample is too small to determine whether medication had any systematic effects on functional connectivity. These data underscore the need for developmental studies of functional connectivity in high- and low-functioning individuals with autism in which head motion is tightly matched between groups.

## Conflict of Interest Statement

The authors declare that the research was conducted in the absence of any commercial or financial relationships that could be construed as a potential conflict of interest.

## Footnotes

1

2

3
